# Benthic Primary Production Budget of a Caribbean Reef Lagoon (Puerto Morelos, Mexico)

**DOI:** 10.1371/journal.pone.0082923

**Published:** 2013-12-18

**Authors:** Malik S. Naumann, Carin Jantzen, Andreas F. Haas, Roberto Iglesias-Prieto, Christian Wild

**Affiliations:** 1 Coral Reef Ecology Group (CORE), Leibniz Center for Tropical Marine Ecology (ZMT), Bremen, Germany; 2 Unidad Academica Puerto Morelos, Instituto de Ciencias del Mar y Limnologıa, Universidad Nacional Autonoma de Mexico (UNAM), Cancun, Quintana Roo, Mexico; 3 Faculty of Biology and Chemistry, University of Bremen, Bremen, Germany; King Abdullah University of Science and Technology, Saudi Arabia

## Abstract

High photosynthetic benthic primary production (P) represents a key ecosystem service provided by tropical coral reef systems. However, benthic P budgets of specific ecosystem compartments such as macrophyte-dominated reef lagoons are still scarce. To address this, we quantified individual and lagoon-wide net (P_n_) and gross (P_g_) primary production by all dominant functional groups of benthic primary producers in a typical macrophyte-dominated Caribbean reef lagoon near Puerto Morelos (Mexico) via measurement of O_2_ fluxes in incubation experiments. The photosynthetically active 3D lagoon surface area was quantified using conversion factors to allow extrapolation to lagoon-wide P budgets. Findings revealed that lagoon 2D benthic cover was primarily composed of sand-associated microphytobenthos (40%), seagrasses (29%) and macroalgae (27%), while seagrasses dominated the lagoon 3D surface area (84%). Individual P_g_ was highest for macroalgae and scleractinian corals (87 and 86 mmol O_2_ m^−2^ specimen area d^−1^, respectively), however seagrasses contributed highest (59%) to the lagoon-wide P_g_. Macroalgae exhibited highest individual P_n_ rates, but seagrasses generated the largest fraction (51%) of lagoon-wide P_n_. Individual R was highest for scleractinian corals and macroalgae, whereas seagrasses again provided the major lagoon-wide share (68%). These findings characterise the investigated lagoon as a net autotrophic coral reef ecosystem compartment revealing similar P compared to other macrophyte-dominated coastal environments such as seagrass meadows and macroalgae beds. Further, high lagoon-wide P (P_g_: 488 and P_n_: 181 mmol O_2_ m^−2^ lagoon area d^−1^) and overall P_g_:R (1.6) indicate substantial benthic excess production within the Puerto Morelos reef lagoon and suggest the export of newly synthesised organic matter to surrounding ecosystems.

## Introduction

Tropical coral reefs are among the most productive global ecosystems on account of a diverse community of benthic photoautotrophs sustaining reef community biomass and ecosystem productivity [1]. For an entire coral reef ecosystem, composed of various physiographic zones (e.g., reef lagoon, reef flat and outer reef slope), gross primary production (P_g_) and respiration (R) appear largely balanced (P_g_:R 1.07) [2], [3]. Consequently, net primary production (P_n_) is low, and hardly any excess production or export of organic matter occurs on the ecosystem scale [4]–[6]. This balanced energetic budget in oligotrophic reef-surrounding waters is driven by the efficient utilization and regeneration of organic and inorganic nutrients via tightly coupled biogeochemical element cycles [7]–[10]. From a more detailed perspective, particular reef ecosystem compartments (e.g., lagoon or reef flat) show considerable differences in benthic primary production (P) and R, which in turn underlie considerable variability [8]. This results from variable contributions of the main functional groups of photoautotrophic primary producers (e.g., seagrasses, macroalgae and scleractinian corals) to benthic community composition, but also from their individual metabolic activity [2], [3].

In contrast to entire coral reef ecosystems and reef compartments dominated by scleractinian corals, there is still very few information available on benthic metabolism and P budgets of macrophyte-dominated reef compartments such as sandy lagoons, seagrass meadows or macroalgae beds [11]–[14]. In addition, the majority of fundamental key studies on the metabolism of tropical coral reefs and their associated coastal habitats is relatively old [1], [15], and thus may not generally suit to address the present state of shifting and/or degraded benthic reef communities observed world-wide over the past decades [16]–[18], calling for a contemporary reassessment.

Most previous studies assessing ecosystem metabolism to generate benthic P budgets in tropical coastal environments have quantified bulk metabolic fluxes on the ecosystem scale using flow respirometry [5], [19], [20]. Other studies have focussed on the physiology of specific benthic primary producers [11], [14], [21], [22] without taking into account the full range of individual contributions to ecosystem productivity within the diverse community of benthic photoautotrophs. However, for reliable benthic P budget calculations, individual P and R of all dominant benthic primary producers and their specific contributions to ecosystem P need to be quantified. In addition, reliable 3D surface area estimates of all investigated organisms, as ecological interface with the surrounding environment, are of paramount importance for the quantification of individual metabolic rates and the calculation of benthic P budgets on ecosystem scale [23], [24].

The goals of this study therefore were to (1) characterise the benthic community composition in a Caribbean fringing reef lagoon and identify the dominant benthic primary producers, (2) quantify individual P and R for all dominant primary producers and their respective functional groups based on estimates of actual 3D surface area, and hence (3) generate a benthic P budget for a macrophyte-dominated tropical coral reef lagoon.

## Materials and Methods

### Study site

**Figure 1:**
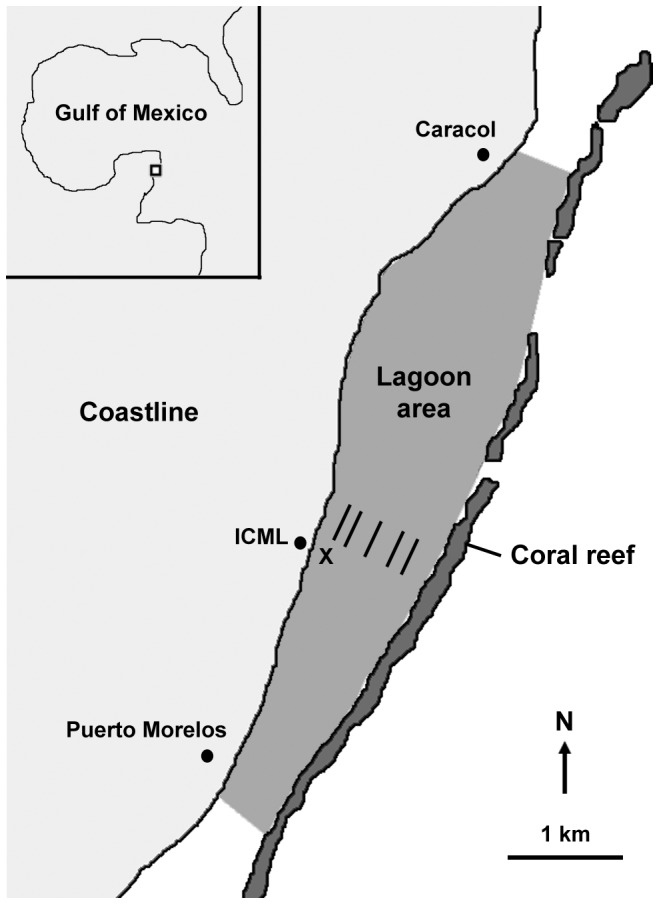
Map of the study site displaying the Puerto Morelos reef lagoon (Mexico) and the total lagoon area considered for primary production budget calculations. The monitoring station for data logger deployments is indicated by X. Parallel lines represent locations of benthic transect surveys. Abbreviation: ICML = Institute of Marine Sciences and Limnology.

This study was carried out from July 15^th^ to August 2^nd^ 2008 (Caribbean summer) in a semi-enclosed Caribbean coral reef lagoon near Puerto Morelos, Mexico (20°52. 058 N, 86°52.030 W) bordering the north–south orientated Mesoamerican barrier reef system (Figure 1). Field work was conducted under permits issued by the Secretaría de Agricultura, Ganadería, Desarrollo Rural, Pesca y Alimentacion to the National Autonomous University of Mexico (UNAM). All measurements were conducted on site in the aquarium and laboratory facilities of the Institute of Marine Sciences and Limnology (ICML) belonging to UNAM.

The coral reef ecosystem of Puerto Morelos features an extensive reef lagoon framed by the coastline and the barrier reef at widths ranging from 0.2 to 1.5 km [25]. Central parts of the lagoon reach maximum water depths of ca. 4 m. Mean residence time for the entire lagoon water body is ca. 3 h and current speeds average 0.1 m s^−1^ under calm conditions [26]. The reef lagoon is affected by seasonal variability in water temperature (up to 5 °C) and light availability due to differences in daytime duration (ca. 2.5 h) between summer maxima and winter minima [27].

Line point intercept surveys (LPI) were conducted to quantify benthic lagoon community composition and identify dominant benthic primary producers. In total, five LPI of 100 m length each were carried out for various distances from land (100, 200, 400, 800 and 1000 m) along a transect perpendicular to the shore-line in front of the ICML (Figure 1). The transect location was selected to include all major lagoon zones (i.e., coastal, mid-lagoon and back reef) to be representative of the lagoon-wide mean seafloor coverage by all dominant benthic functional groups and substrate types. The representative quality of the transect location was confirmed by previous studies on lagoon-wide spatial macroalgae biomass distribution and mean seagrass shoot density by dominant benthic macrophytes (i.e., *Halimeda incrassata, Thalassia testudinum*) [28]–[30]. Intervals of 0.5 m between intercepts resulted in 201 data points per LPI. Results obtained from all LPI were used to calculate the lagoon-wide mean percentage coverage for each recorded benthic taxon or substrate type. The 2D area of the entire reef lagoon (ca. 8.1423 km^2^) extending between shore-line locations at the level of the town centres of Puerto Morelos and Caracol (located northerly, Figure 1) was determined from an aerial digital photograph using image processing software (ImageJ, V. 1.37 m, National Institutes of Health).

### Sample collection and maintenance

Specimens representing the major functional groups of primary producers (i.e., seagrasses, macroalgae, sand-associated microphytobenthos and scleractinian corals) were collected from similar intermediate water depths (2.0–2.5 m) using SCUBA. From each functional group, the main representative taxa were collected: 1) seagrasses: *Thalassia* and *Syringodium*, 2) macroalgae: green (*Halimeda* and *Avrainvillea*), brown (mainly *Lobophora*) and several unidentified red algae taxa, 3) scleractinian corals: *Porites* and *Manicina*, 4) microphytobenthic mixed sand community: not identified taxonomically. This selection of organisms together accounted for 95% of the 2D lagoon seafloor coverage and for 98% of all benthic primary producers. Collection techniques and post-sampling treatment for the respective organisms are described below.

A flow-through cultivation tank (volume: 200 L) supplied with freshly pumped lagoon water (exchange rate: 150–200 L h^−1^) was set up for the collected seagrass, macroalgae and coral specimens to heal under *in situ* temperature (study period range: 29–32°C) and light conditions (mid-day photosynthetically active radiation ca. 600 µmol quanta m^−2^ s^−1^) before experimentation. Both, temperature and light availability were continuously recorded during the study period by data loggers (Onset HOBO Pendant UA-002-64, n = 3) in the cultivation and experimental tanks and in the lagoon at a reference site (water depth: 2.5 m, 1 min resolution, Figure 1). To ensure that conditions were congruent throughout the entire study period, data loggers were initially deployed for 2 d prior to the first transfer of organisms to the cultivation tank, and thereafter analysed and redeployed every second day. Light availability in the cultivation and experimental tanks (daytime mean±SD: 17,161±16,270 lux) was adjusted to *in situ* lagoon conditions using net cloth, while water temperature was controlled by regulating flow-through rates.

Branching colonies of *Porites* (diameter 7–9 cm, n = 12) and solitary *Manicina* polyps (diameter: 3–9 cm, n = 12) were sampled from the lagoon seafloor 1 wk prior to measurements. All coral specimens were collected and transferred using individual zip lock bags to avoid mechanical damage during transport. In the lab, corals were fixed onto ceramic tiles using epoxy glue (Reef Construct, Aqua Medic) to avoid direct tissue contact during handling. During the recovery period, ceramic tiles were regularly cleaned from overgrowth. Seagrasses (height: 12–26 cm, n = 12 for both taxa) and benthic macroalgae specimens (height: 5–12 cm, n = 12 for each of 4 taxa) were collected 48 h prior to physiological measurements and left to heal. Lagoon sand samples (n = 12) were obtained using a custom-made “mini-corer” apparatus with a defined 2D surface area (5.73 cm^2^), volume (3.4 ml) and sediment core depth (1.5 cm). These samples were immediately transported to the laboratory for subsequent quantitative net primary production (P_n_) and respiration (R) measurements of the associated microphytobenthos. During handling, special care was taken to transfer all specimens without air exposure and to exclude seagrasses, macroalgae and corals showing extensive epibiont or endolith infestations potentially affecting O_2_ fluxes during incubation experiments.

### Surface area quantification

Digital photographs of spread out macroalgae and seagrasses were used to quantify the 2D leaf surface area by image processing software (ImageJ, V. 1.37 m, National Institutes of Health). The planar area of each specimen was subsequently multiplied by the factor 2 to obtain the 3D surface area representing both sides of the outspread macrophytes. The 3D surface area of each macroalgal and seagrass specimen was related to the respective 2D lagoon area at the sand-water interface determined during LPI surveys to generate the 2D to 3D area conversion factor for each taxon. Skeletal 3D surface area of scleractinian corals was quantified using the Advanced Geometry technique with subsequent application of coral growth form specific approximation factors [31]. Estimates for skeletal 3D surface area and planar projected surface areas of scleractinian coral specimens, likewise quantified by digital image analysis, were used to generate 2D to 3D surface area conversion factors for both coral taxa. The specific resulting 2D to 3D conversion factors are summarised in Table 1. For samples of the microphytobenthic sand community, specimen surface area was represented by the sampled 2D area defined by the “mini-corer” apparatus (cf. Sample collection and maintenance).

**Table 1:** Individual primary production and respiration rates measured for all dominant benthic primary producers in a Caribbean reef lagoon (Puerto Morelos, Mexico).

Functional group	Genus	P_g_	R	P_n_	2D:3D	n
		(mmol O_2_ m^−2^ specimen area d^−1^)				
**Seagrasses**	*Syringodium*	24±9	15±9	11±8	80.5±7.3	6
	*Thalassia*	13±3	11±5	4±2	46.3±3.5	6
	**Mean**	**18**±**9**	**13**±**7**	**8**±**7**		
**Macroalgae**						
Green algae	*Halimeda*	75±9	32±9	42±9	13.3±3.7	6
	*Avrainvillea*	52±5	25±5	27±5	14.0±2.2	6
	Mean	63±14	29±8	35±11		
Brown algae	*Lobophora*	40±7	17±3	23±7	2.5±0.1	6
Red algae	unidentified	182±31	89±25	93±31	1.3±0.1	6
	**Mean**	**87**±**60**	**41**±**32**	**46**±**33**		
**Microphytobenthos**	unidentified	**46**±**10**	**34**±**8**	**16**±**10**	1.0	6
**Scleractinian corals**	*Manicina*	91±10	79±29	12±10	1.6±0.1	6
	*Porites*	81±13	72±10	16±9	2.3±0.1	6
	**Mean**	**86**±**12**	**74**±**19**	**14**±**9**		

_g_, R and P_n_ are given as mean±SD, 2D:3D factors as mean±SE. Abbreviations: P_g_ = gross primary production, P_n_ = net primary production, R = respiration, n = number of replicates. Values for P

### Physiological measurements

To quantify individual P_n_ and R rates via O_2_ fluxes, specimens were incubated with fresh lagoon seawater inside gas-tight glass chambers (500 mL), which were submerged into a 200 L flow-through experimental tank serving as a water bath. The temperature and light conditions in the experimental tank were adjusted to be identical to those of the cultivation tank and congruent to *in situ*. For each taxon-specific incubation experiment, one set of specimens (n = 6) was incubated in the light (P_n_), another set (n = 6) in the dark (R), while 8 additional chambers containing only lagoon seawater served as seawater controls for P_n_ (n = 4) and R (n = 4) incubations. R treatment and seawater control incubation chambers were covered using an opaque plastic foil preventing ambient light penetration. All chamber incubations were started at ca. 10∶00 to minimise potential effects of potential circadian rhythms and, except for scleractinian corals, lasted for 24 h to obtain representative diel rates for P_n_. For scleractinian corals, the incubation period was reduced to 6 h to prevent physiological damage by hypoxic or hyperoxic conditions. Water flow velocities inside the incubation chambers could not be accurately adjusted to flow conditions comparably low as in situ (mean: 0.1 m s^−1^), without risking substantial overestimation of physiological rates by elevated flow velocities [32]–[36]. Thus, all chamber incubations were conducted under no-flow conditions to generate conservative estimates for benthic primary producer P_n_ and R rates. No-flow conditions ensured higher measurement accuracy of P_n_ and R rates ruling out flow-induced effects on O_2_ transfer velocities across the surface boundary of the incubation chambers [37], while also allowing for comparison with previous chamber incubation studies [24], [38]. At the start and end of all chamber incubation, the concentration of dissolved O_2_ (DO) was measured in the incubation medium using a DO optode sensor (Hach Lange, HQ10) after gently stirring for 5 s before every reading to ensure homogeneous DO distribution.

### Data analyses

P_n_ and R for each incubated specimen were derived from DO concentration differences calculated by subtracting start from end concentrations. These results were corrected for DO concentration differences measured in seawater controls and normalised to incubation volume, specimen surface area and incubation period. Diel rates of P_n_ and R for both scleractinian coral taxa were calculated by extrapolation of 6 h incubation periods to an approximate 12∶12 h light/dark cycle (07:00–19:00) measured *in situ* during the study period. Individual gross primary production (P_g_) rates were calculated by adding mean R rates for each specific taxon to the individual P_n_ of each incubated specimen. This resulted in individual P_g_, R and P_n_ rates for all incubated taxa (given as: mmol O_2_ m^−2^ specimen surface area d^−1^).

Individual P_g_, R and P_n_ rates were used to calculate the contribution of each investigated taxon and their respective functional groups to lagoon-wide benthic metabolism taking into account the specific percentage cover related to the entire lagoon area (ca. 8.1423 km^2^, Figure 1) and respective mean 2D to 3D conversion factors. To account for the notable contribution (11% of green algae cover) by unidentified taxa of the diverse benthic green algae community [39], mean metabolic rates and conversion factors derived from *Halimeda* and *Avrainvillea* were applied. Resulting taxon- and functional group-specific contributions to lagoon-wide P_g_, R and P_n_ were normalised to seafloor area by the total 2D lagoon area and expressed as mmol O_2_ m^−2^ lagoon area d^−1^. Individual and lagoon area-related taxon- and functional group-specific P_g_, R and P_n_ data sets were analysed statistically using one-way ANOVA with Tukey or Games-Howell *post-hoc* tests (SPSS software packages, v. 14.0, IBM), if not mentioned differently, after testing for equal variances (Levene test) and normal distribution (Kolmogorov–Smirnov test).

## Results

### Benthic community composition

**Figure 2:**
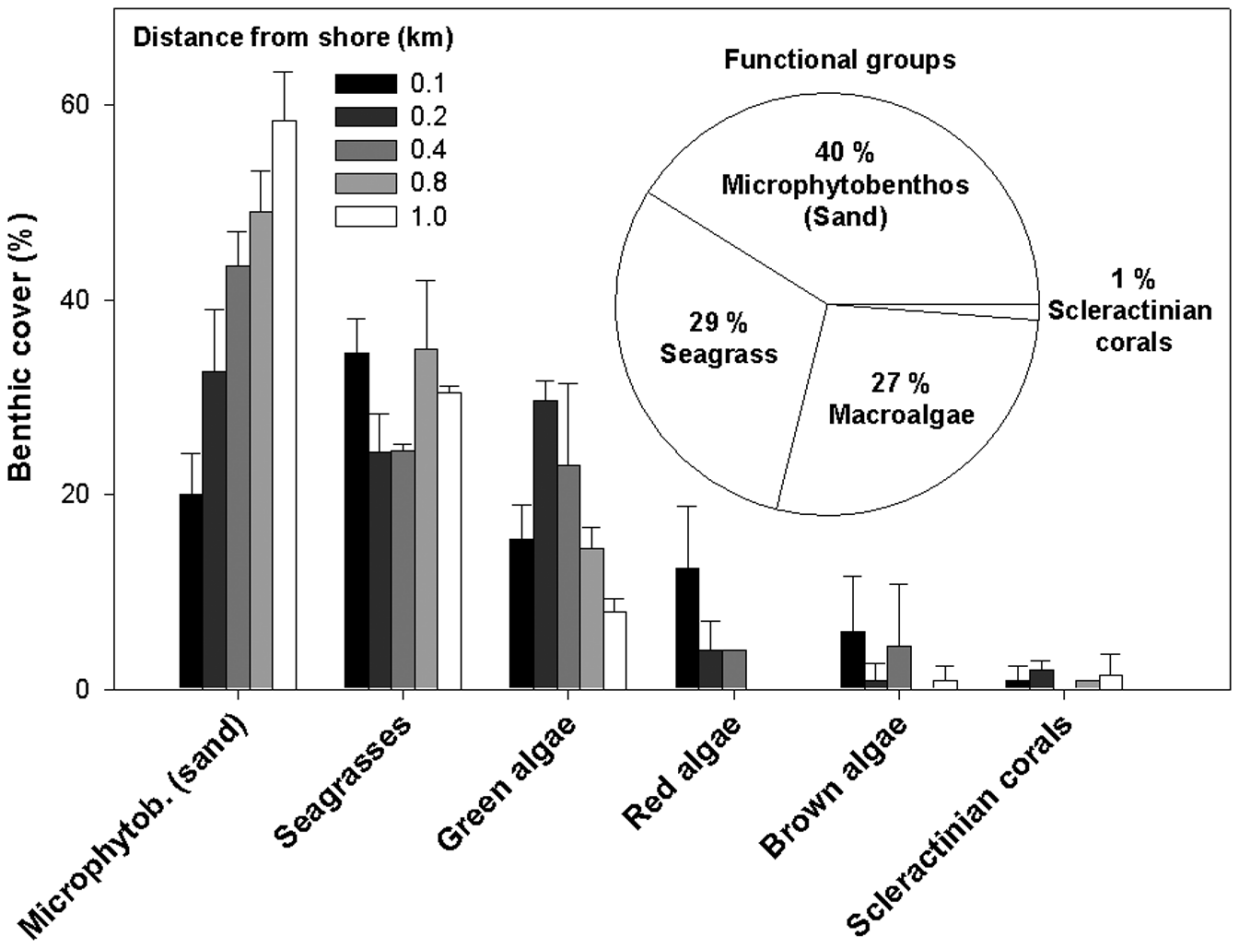
Benthic coverage by dominant primary producers in the Puerto Morelos reef lagoon (Mexico). Bar chart shows percentage cover of the lagoon 2D area by the monitored categories, sand (microphytobenthos), seagrasses (*Thalassia* and *Syringodium*), green algae (*Avrainvillea, Halimeda, Rhipocephalus, Penicillus* and other unidentified taxa), red algae (unidentified), brown algae (mainly *Lobophora*) and scleractinian hard corals (*Porites* and *Manicina*) by increasing distance to shore. Values are given as mean±SD. Pie chart indicates the lagoon-wide mean percentage cover by the respective functional groups.

**Figure 3:**
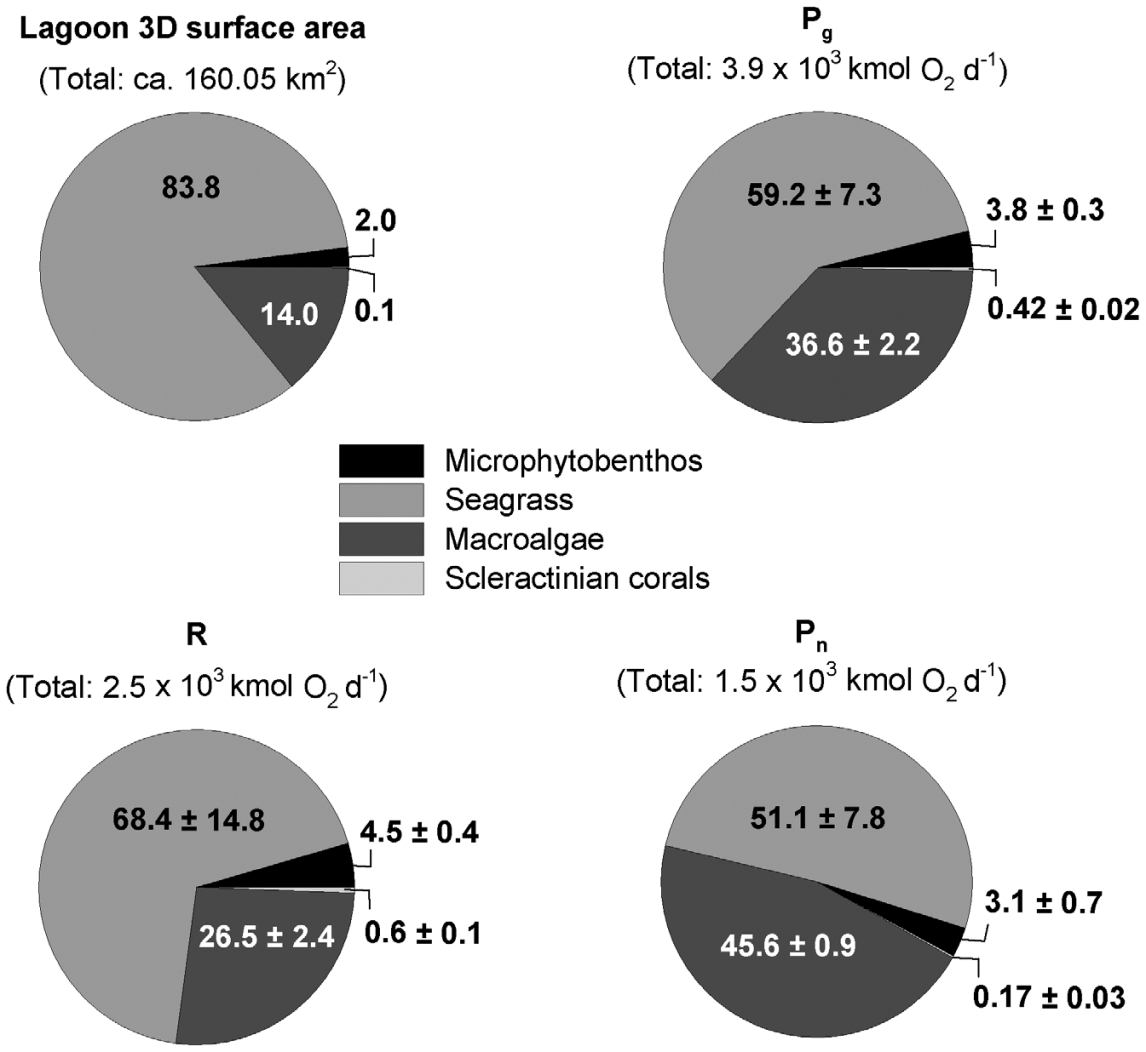
Benthic primary production budget for a Caribbean reef lagoon (Puerto Morelos, Mexico). Values in pie-chart slices are given as percentage mean±SE of percentage contribution to 3D lagoon surface area, primary production or respiration. Abbreviations: P_g_ = gross primary production, P_n_ = net primary production, R = respiration.

In terms of 2D lagoon areal coverage, calcareous sand accounted for most of the seafloor area (mean: 40%), and thus sand-associated microphytobenthos represented the spatially dominant functional group (Figure 2). However, after 2D to 3D surface area conversion including all functional groups and substrate types (total 3D lagoon surface area: 160.05 km^2^), the percentage contribution by microphytobenthos was minor (Figure 3). Seagrasses (29.2%) and macroalgae (26.8%) occupied similar considerable fractions of the 2D lagoon area, which converted into the major (83.8%) and second (14.0%) largest share of 3D lagoon surface area, respectively. In contrast, scleractinian coral cover was minimal for 2D (1.2%) and 3D (0.1%) lagoon surface area estimates.

### Metabolic rates of benthic primary producers

Scleractinian corals and macroalgae showed the highest (F_3,50_ = 8.87, p<0.018), yet similar (p = 1.00), individual gross primary production (P_g_) of all functional groups, while scleractinian corals exhibited the highest individual respiration (R) (F_3,50_ = 12.33, p = 0.01) and seagrasses the lowest individual R (p<0.002) (Table 1). Net primary production (P_n_) was highest for macroalgae (F_3,50_ = 8.38, p<0.04) and similar for seagrasses, microphytobenthos and scleractinian corals (p>0.90).

Within functional groups, P_g_ was similar for the scleractinian coral genera *Manicina* and *Porites* (t-test, t = 1.54, df = 10, p = 0.155), while amongst macroalgae P_g_ rates were highest for red and lowest for brown algae (p<0.001, p<0.029; Table 1). P_g_ of the seagrass *Syringodium* was elevated compared to *Thalassia* (Mann-Whitney U-test, *z* = -2.24, p = 0.03). The overall highest individual R rates were measured for red algae (F_3,50_ = 28.76, p<0.025), which were however similar to R of both coral taxa (p>0.77). Individual R rates of both seagrasses were similar (t-test, t = 4.94, df = 10, p = 0.31). P_n_ was similar for both investigated scleractinian corals (t-test, t = −0.56, df = 10, p = 0.59) and among seagrasses (Mann-Whitney U-test, z = −2.24, p = 0.06), while red algae showed the highest P_n_ of all investigated macroalgae taxa (p<0.028).

### Benthic lagoon primary production budget

Seagrasses exhibited the highest lagoon area-related P_g_ compared to all other functional groups (F_3,50_ = 42.48, p<0.001), while contributing the largest share (59.2%) to lagoon-wide P_g_ (Table 2, Figure 3). P_g_ of seagrasses was substantially elevated compared to R (P_g_:R = 1.4±0.2, mean±SE), resulting in the likewise highest contribution (51.1%) to lagoon-wide P_n_ (Figure 3). Macroalgae P_g_ related to lagoon area was higher compared to microphytobenthos (p = 0.036) and scleractinian corals (p<0.001), and contributed the second largest share to lagoon-wide P_g_ (Figure 3). Macroalgae R was only less than 50% of macroalgae P_g,_ resulting in the second highest area-related P_n_ of all investigated functional groups (Table 2). High individual macroalgae P_n_ rates were reflected in the second largest contribution (45.6%) to lagoon-wide P_n_ (Figure 3). In contrast, microphytobenthos contributed only minor fractions to lagoon-wide P_g_ and R, eventually accounting for only 3.1% of lagoon-wide P_n_, as a result of low 3D area coverage. Area-related P_g_ and R rates for both scleractinian corals were nearly balanced and lower than for all other functional groups (F_3,50_ = 42.48, p<0.001; F_3,50_ = 33.72, p<0.007, respectively), likewise showing extremely low P_n_ rates, and consequently negligible contributions to lagoon-wide metabolism (Figure 3).

**Table 2:** Primary production and respiration rates of all dominant benthic primary producers normalised to lagoon seafloor area.

Functional group	Genus	P_g_	R	P_n_	P_g_:R
		(mmol O_2_ m^−2^ lagoon area d^−1^)			
**Seagrasses**	*Syringodium*	164±62	106±61	74±54	1.6±0.3
	*Thalassia*	125±25	104±51	34±18	1.2±0.3
	**Mean**	**144**±**50**	**105**±**53**	**57**±**45**	**1.4**±**0.2**
	**Sum**	**289**±**88**	**210**±**111**	**109**±**72**	
**Macroalgae**					
Green algae	*Halimeda*	85±10	37±11	48±10	2.3±0.3
	*Avrainvillea*	62±6	30±6	32±6	2.1±0.1
	Mean	69±20	32±11	38±14	2.2±0.1
Brown algae	*Lobophora*	4±1	1.6±0.3	2±1	2.3±0.1
Red algae	unidentified	10±2	5±1	5±2	2.0±0.1
	**Mean**	**39**±**35**	**18**±**17**	**21**±**20**	**2.2**±**0.2**
	**Sum**	**178**±**18**	**81**±**18**	**97**±**18**	
**Microphytobenthos**	unidentified	**19**±**4**	**14**±**3**	**7**±**4**	**1.3**±**0.1**
**Scleractinian corals**	*Manicina*	0.5±0.1	0.5±0.2	0.07±0.06	1.2±0.2
	*Porites*	1.5±0.2	1.3±0.2	0.3±0.2	1.1±0.1
	**Mean**	**1.0**±**0.5**	**1.0**±**0.5**	**0.162**±**0.159**	**1.0**±**0.1**
	**Sum**	**2.0**±**0.3**	**1.8**±**0.4**	**0.4**±**0.2**	
**Total**		**488**±**59**	**307**±**94**	**181**±**28**	
**Mean**					**1.6**±**0.4**

_g_, R and P_n_ are given as mean±SD. P_g_:R ratios are presented as mean±SE. Abbreviations: P_g_ = gross primary production, P_n_ = net primary production, R = respiration. Values for P

## Discussion

### Budget of benthic lagoon primary production

This study represents one of the very few field investigations succeeding early fundamental works [1] that generates a benthic primary production (P) budget for a coral reef-associated habitat (i.e., reef lagoon) based on individual metabolic rates of all dominant benthic primary producers. To our knowledge, it is the first to calculate a benthic P budget by combining taxon-specific metabolic rates with the respective estimates of photosynthetic 3D surface areas. Our findings characterise the investigated Caribbean reef lagoon as a net autotrophic and macrophyte-dominated benthic environment, in which seagrasses and macroalgae account for the main fractions of 2D (i.e., seagrasses + macroalgae) and 3D (seagrasses) benthic lagoon area. P budget calculations based on individual gross primary production (P_g_), net primary production (P_n_) and respiration (R) rates identify seagrasses and benthic macroalgae as the major contributors to lagoon-wide high photosynthetic P_n_. Considerable excess P by benthic photoautotrophs suggests rapid turnover by microbial degradation in lagoon overlying waters or the potential export from this macrophyte-dominated coral reef ecosystem compartment.

As the present study was conducted exclusively during Caribbean summer season, our findings for individual metabolic rates may be elevated compared to other seasons and to annual average. P of benthic primary producers may be influenced by seasonal variations in key environmental parameters, such as water temperature and light availability [27], [29], [30]. However, all investigated seagrass and macroalgae taxa, the major contributors to lagoon area coverage, show no obvious seasonal dynamics in biomass and seafloor coverage [27]. This indicates the annual validity of our summer lagoon area coverage data and further suggests a similar percentage contribution by the respective primary producers to the lagoon P budget over a yearly cycle. However, as all chamber incubations were conducted under no-flow conditions, individual metabolic rates are expected to be higher under in situ flow conditions [32]–[36], and may eventually cause a potential increase of the calculated lagoon-wide P.

With respect to the high 2D benthic cover by macrophytes and the substantial contribution by seagrasses to lagoon-wide photosynthetic 3D surface area, our present findings for metabolic rates can most adequately be compared to tropical coastal habitats featuring seagrass meadows. This is supported by our findings for area-related lagoon-wide P_g_, P_n_ and R, which fall into the upper range of metabolic rates recently reviewed and summarised for tropical seagrass meadows [13] that likewise show identical mean P_g_:R ratios (Table 3). Further, our area-related P_g_, P_n_ and R for both investigated seagrass taxa are similar to mean metabolic rates listed for monospecific meadows likely dominated by the same seagrass species, or at least same genus, investigated here (i.e., *Thalassia testudinum* and *Syringodium filiforme*) [13]. Consistent with our findings for the Puerto Morelos reef lagoon, most seagrass ecosystems tend to be net autotrophic [13], [40], [41] showing pronounced P_n_ similar to other macrophyte-dominated ecosystems, such as mangroves forests or macroalgae beds (Table 3). Previous work in the study site has shown that this net productive state is even largely sustained during and after severe disturbances such as tropical storm events with substantially reduced ambient seawater temperature and light availability [42]. Further, our results for lagoon-wide P_g_ related to seafloor area are in the range of established literature values for entire reef ecosystems, while lagoon-wide P_n_ rates are even approximately one order of magnitude higher (Table 3). This in turn suggests a substantial input by the lagoon compartment to ecosystem-wide P in tropical coral reefs, which eventually may dependent on the areal contribution of the lagoon to the entire reef ecosystem area.

**Table 3:** Overview of benthic primary production in coral reef ecosystem compartments and reef-associated tropical coastal habitats.

Benthic ecosystem	Region	P_g_	P_n_	P_g_:R	Source
		(mmol O_2_ m^−2^ seafloor area d^−1^)			
**Coral-dominated**					
Reef flats	various Pacific	583±50		1.0±0.1	[4]
Entire reefs	various Pacific	395±58	27±20	1.07±0.1	[3]
**Macrophyte-dominated**					
Reef lagoon	Caribbean	488±59	181±28	1.6±0.4	This study
Seagrass meadows	Tropical	252±14	24±8	1.6	[13]
Macroalgae beds	Central Pacific	520		1.04	[14]
	Caribbean	877	582	3.0	[11]
**Sediments**					
Lagoon sand	Caribbean	19±4	7±4	1.3±0.1	This study
	Northern Red Sea	19±4	−1±2	0.99	[12]
Lagoon soft sediments	SW Pacific	33		0.88	[45]
Reef sand	SC Pacific	91	7	1.07	[21]
					
**Mangroves**	mainly Caribbean	633±117	400±83	1.4±0.4	[3]
**Estuaries**	various Atlantic and Pacific	67±8	−17±8	0.8±0.1	[3]

_g_ and P_n_ are presented as means±SE. If necessary, original units were converted to O_2_ estimates assuming PQ = RQ = 1 [13], [44]. Abbreviations: P_g_ = gross primary production, P_n_ = net primary production, PQ = photosynthetic quotient, RQ = respiratory quotient. Values for P

Seagrasses and macroalgae contribute the major fractions to P_g_, P_n_ and R in the Puerto Morelos reef lagoon. Notably, seagrasses account for the largest lagoon-wide shares, although their individual metabolic rates are lower than of the local benthic macroalgae community covering comparable fractions of 2D lagoon area. This apparent inconsistency clearly results from the dominance of seagrasses regarding lagoon-wide photosynthetic 3D surface area, quantified here for the first time by the generation of taxon-specific 2D to 3D conversion factors. However, high seagrass R likewise affects the lagoon-wide P_n_ shares of all other functional groups, thus revealing the substantial contribution of benthic macroalgae to the production of newly photosynthesised organic matter. This finding supports a recent study describing the dynamic growth of the benthic macroalgae community in this reef lagoon, thereby rapidly spreading over newly available benthic substrate generated by environmental disturbances [27].

Microphytobenthos and scleractinian corals represent the functional groups with low to insignificant contributions to lagoon-wide P_g_, P_n_ and R. The very low 2D and 3D areal cover by scleractinian corals determines their insignificant contribution to lagoon-wide metabolism, despite the fact that individual coral P_g_ and R rates are among the highest of all functional groups, and comparable to other Caribbean coral taxa investigated at the study site [43]. Likewise, sand-associated microphytobenthos shows the highest individual P_n_ rates and covers the major fraction of 2D lagoon area, resulting in the third largest share of lagoon-wide P_n_. However, this share appears low due to the disproportionally minor contribution by microphytobenthos to the lagoon-wide 3D surface area. In fact, microphytobenthos P_n_ rates measured at this Caribbean site are substantially higher than described for other shallow coral reef lagoons (Table 3), as in sandy lagoons of the Northern Red Sea [12]. There, microphytobenthos P_g_ and R are balanced (P_g_:R = 0.99), characterising these benthic substrates as largely independent of allochthonous organic matter input, while exhibiting low to no photosynthetic P_n_. Interestingly, P_g_ rates of these Red Sea lagoon sands seem identical to those of the present study site (Table 3), suggesting substantially higher R in Red Sea lagoons, likely sustained by abundant and specialised heterotrophic microbes [46]. Low R of calcareous lagoon sands may indicate a decreased, or regionally variable, intensity of their biocatalytical filter function with potential implications for biogeochemical cycling and the fate of lagoon-wide P_n_.

Benthic P_n_ and P_g_:R of this coral reef-associated lagoon are in the range of previous findings for other macrophyte-dominated coastal habitats, such as mangrove forests or seagrass meadows (Table 3), known for their substantial export of photosynthetic excess production. Substantial amounts of P_n_ (24.3–43.5%) may be exported from macrophyte-dominated benthic communities as detritus, undergo burial processes and microbial decomposition, e.g., in neighbouring beach ecosystems, or enter export to the open ocean [6], [47]–[49]. However, previous work at the study site also indicates that the majority of benthic primary producers, in particular seagrasses and macroalgae, show a constant release of newly photosynthesised P_n_ in the form of dissolved (DOM) and particulate (POM) organic matter, and that this organic matter stimulates microbial O_2_ consumption in local lagoon overlying waters [50]. DOM release rates by benthic macroalgae (3.8 mmol C m^−2^ d^−1^) account for ca. 26% of mean benthic macroalgae P_n_ quantified by the present study, thus ranging within margins of current P_n_ export estimates for macrophyte-dominated benthic communities [6]. Release of DOM and POM with labile biochemical properties and its subsequent decomposition by planktonic microbes may thus initiate short linked benthic-pelagic element cycling between the lagoon compartments similar to that described for sandy Red Sea lagoons [51], [52]. Further integrated laboratory and in situ studies may elucidate the role of this potential trophic pathway and increase our understanding of the biogeochemical processes determining the fate of benthic photosynthetic P_n_ in reef-associated lagoon environments.
